# On Albumen as a Cholagogue

**Published:** 1855-09

**Authors:** R. Gieseler

**Affiliations:** Göttingen


*On Albumen as a Cholagogue.* By Dr. R. Gieseler of Göttingen. —I am anxious to call the attention of the profession, briefly, to the employment of the albumen of hen's eggs in certain forms of jaundice.

Bernard's experiments, showing that this substance is assimilable only through the intervention of the hepatic function, immediately suggested to me the idea that in albumen we might find an adequate excitant of the liver. I inferred, first, that fatty nutriment, and in a higher degree albuminous articles of diet, must be avoided in inflammation of the liver; and secondly, that in torpid conditions of that organ we might possess in albumen a remedy capable of stimulating it to increased activity. If to the liver be assigned the task of rendering albumen adapted to assimilation, this substance must be a stimulant of it, which will, *mutatis mutandis*, set its function to work, in the same manner as the administration of saline medicines does that of the kidneys. It is scarcely necessary to add, that the establishment of these results by experience must secure to albumen not merely the character of an adequate stimulant, but also pre-eminence over all other so-called cholagogues, since the action of the latter is very uncertain.

I think it unnecessary to demonstrate the remarkable efficacy of albumen in this respect by the recital of cases, since it was, as I soon learned, already known to our predecessors. It, however, appears to me not unimportant to point out the source whence it would appear the recommendation to employ albumen as a remedy in jaundice was originally derived. Charles White, in his work on "The Treatment of Pregnant and Puerperal Women," states, that he once suffered for several weeks from jaundice, and was very much reduced. Soap, aloes, iron, and rhubarb, had been taken without the least benefit. A navy officer, happening to visit him, assured him he would cure him in a short time. He told him, in fact, that while on a voyage some time before, he was attacked with the same disease, and had in vain used the remedies prescribed by the surgeon of the vessel. A Spanish physician of the island of Minorca then advised him to take every morning, while fasting, two raw eggs, both yolk and white, in a glass of water, and to repeat the dose with one egg every four hours during the day. He followed this advice, and in three days his motions were again colored with bile. White tried the plan suggested, and found the effect attributed to the albumen to be confirmed; in three days the fæces were colored, which they had not been for six weeks before. He continued the use of the eggs for some months. He subsequently recommended the remedy to several patients, and always with good effect, except in cases in which the jaundice preceeded from the presence of gallstones. So far for the testimony of Mr. White. In the more modern treatises on therapeutics I have not been able to find any allusion to this application of albumen; the present communication cannot, therefore, be considered superfluous. A few of the older works recommend, not white of egg, but the yolk, probably on account of its yellow color. It is indeed possible that the action of the liver may be excited, not by the vitellin of the yolk, but merely by the albumen of the egg, with which Bernard experimented, and which White recommended in jaundice. Should this supposition prove correct, it would explain why the remedy lapsed into oblivion, and would furnish an important proof in our day for the often misunderstood truth, that practical results do not become the property of science or art, until they are referred to correct principles.—*Dublin Quarterly Jour. from Zeitschrift fur Rationelle Medicin*, 1854.

